# Microwave Breast Imaging System Prototype with Integrated Numerical Characterization

**DOI:** 10.1155/2012/706365

**Published:** 2012-03-08

**Authors:** Mark Haynes, John Stang, Mahta Moghaddam

**Affiliations:** Applied Physics Program, Basic Radiological Sciences Ultrasound Group, and Radiation Laboratory, Department of Electrical Engineering and Computer Science, University of Michigan, Ann Arbor, MI 48109-2122, USA

## Abstract

The increasing number of experimental microwave breast imaging systems and the need to properly model them have motivated our development of an integrated numerical characterization technique. We use Ansoft HFSS and a formalism we developed previously to numerically characterize an *S*-parameter- based breast imaging system and link it to an inverse scattering algorithm. We show successful reconstructions of simple test objects using synthetic and experimental data. We demonstrate the sensitivity of image reconstructions to knowledge of the background dielectric properties and show the limits of the current model.

## 1. Introduction

A number of experimental systems for microwave breast imaging have been developed in recent years. These systems test full-wave inverse scattering algorithms [1–4] as well as synthetic aperture beam focusing techniques [5]. While imaging algorithms abound in the literature, techniques to properly model, characterize, and calibrate these systems have lagged behind algorithm development. Investigators have started to identify characterization as a major task, which must be addressed in order to fully evaluate the efficacy of microwave imaging for breast cancer detection. Part of this evaluation involves separating modeling errors from intrinsic algorithm artifacts in the final images. Thus, there is a need for accurate models of experimental systems, as well as methods that efficiently incorporate these models into the imaging algorithms.

The task of characterizing a microwave breast imaging system for inverse scattering, as compared to a free-space system, is complicated by several factors. Specifically, the antennas are not isolated in the background media but exist as part of the surrounding structure. Also, compact arrangements of many antennas create a cavity-like imaging geometry, and the transmitter incident fields include all background multiple scattering. Finally, the antennas and object are in each others near-fields, so object-cavity scattering should be modeled.

In trying to characterize breast imaging systems, investigators have turned to full numerical simulation. The antenna cavity in [6] was modeled using Ansoft HFSS and only used for antenna design and sensitivity analysis. In [7], dipole sources of an inverse scattering experiment were modeled with HFSS and calibration constants used to scale the antenna incident fields. HFSS has also been used to obtain antenna incident fields in a near-field and open, antenna setup [8]; however, ad hoc methods have been used to calibrate the scattered field *S*-parameter data for the inverse scattering algorithm. In more recent work [9], CST Microwave Studio was used to study and tune antenna performance in a breast imaging cavity. Also, finite-volume time-domain solvers of [10] modeled wide-band antennas for time-domain beam focusing. The most complete work to date is [11], where an FEM forward solver is used to simulate the entire breast in the presence of the antennas, but computational complexity remains a challenge. Despite the growing use of numerical solvers to model breast imaging systems, there has been no clear or formal way of incorporating the results from full-wave numerical models into the imaging algorithms.

The task of characterizing any inverse scattering system can be divided into three parts: (1) determining the incident fields produced by the antennas in the absence of the object, (2) determining the background dyadic Green's function, that is, modeling the interactions between the object and its surroundings if necessary, and (3) linking the volume integrals in the imaging algorithms to measurable transmit and receive voltages. The purpose of this paper is to show how we use HFSS and a formalism we developed in previous work [12] to solve parts (1) and (3) of this characterization problem, in order to make a numerical characterization and inverse scattering algorithm consistent with an *S*-parameter based prototype breast imaging system.

The inverse scattering algorithm we use is the Born iterative method (BIM) with multivariate-covariance cost function [13–15]. This cost function allows us to experimentally choose the regularization parameters based on our prior knowledge of system noise and expected range of permittivities. The forward solver used in the BIM requires the background dyadic Green's function and finding it constitutes part (2) of the characterization problem mentioned above. For convenience we use the lossy free-space dyadic Green's function and give some numerical and experimental justification for this. Fully modeling the multiple scattering between the breast and the imaging structure in the forward solver is not trivial and we discuss it in The Appendix.

We validate our methods with a combination of simulation and experiment. We first present the formalism of [12] in the context of cavity problems. We then explain our experimental setup, which consists of a cylindrical imaging cavity with printed antennas, solid-state switching matrix, and water/oil coupling medium. The HFSS numerical model is presented and the simulation results are compared to those of experiment. We form 3D images of the relative permittivity and conductivity using both HFSS synthetic data and experimental data for simple targets. We also present findings on the sensitivity of image reconstructions to the accuracy of modeling the background electrical properties.

Future work includes continuing the validation of our methodology, experimentally imaging more realistic breast phantoms, designing a hemispherical imaging cavity, investigating practical solutions to modeling the breast-cavity scattering interactions, and developing a clinical imaging system.

## 2. Formulation with Source Characterization

### 2.1. Traditional Volume Integral Equations

The electric field volume integral equation (VIE) for an inhomogeneous distribution of permittivity and conductivity is given by

(1) $$E\left(r\right)=E_{\text{inc}}\left(r\right)+k_{o}^{2}\int \bar{G}\left(r,r^{\prime }\right)\cdot \left(\delta \epsilon \left(r^{\prime }\right)+\frac{i\delta \sigma \left(r^{\prime }\right)}{\epsilon_{b}\omega }\right)E\left(r^{\prime }\right)dV\prime ,$$

where **E**(**r**) and **E**_inc_(**r**) are the total and incident fields, respectively, and **r** is the position vector. The lossless background wave number is given by *k*_*o*_^2^ = *ω* ^2^ *μ*_*o*_ *ϵ*_*b*_, where the background permittivity is *ϵ*_*b*_ = *ϵ*_*o*_ *ϵ*_*rb*_ with relative permittivity *ϵ*_rb_. The object contrast functions are defined:

(2) $$\begin{array}{c} \epsilon_{b}\delta \epsilon \left(r\right)=\epsilon \left(r\right)-\epsilon_{b},\\ \delta \sigma \left(r\right)=\sigma \left(r\right)-\sigma_{b}, \end{array}$$

where *σ*_*b*_ is the background conductivity. The quantity *δϵ*(**r**) is unitless and *δσ*(**r**) is an absolute measure of conductivity with units of Siemens per meter and $\bar{G}(r,r\prime )$ is the background dyadic Green's function.

Defining the scattered field as

(3) $$E_{\text{sca}}\left(r\right)=E\left(r\right)-E_{\text{inc}}\left(r\right)$$

and restricting the observation point **r** to points outside the object region in (1), we can write the VIE for the scattered field concisely as

(4) $$E_{\text{sca}}\left(r\right)=\int \bar{G}\left(r,r^{\prime }\right)\cdot O\left(r^{\prime }\right)E\left(r^{\prime }\right)dV^{\prime },$$

where we define the following object function:

(5) $$O\left(r\right)=k_{o}^{2}\left(\delta \epsilon \left(r\right)+i\frac{\delta \sigma \left(r\right)}{\epsilon_{b}\omega }\right).$$

In the context of inverse scattering, (1) represents the solution to the wave equation in the object domain, while (4) relates the material contrasts to scattered field measurements taken outside the object domain. Depending on the inversion algorithm, these two equations are used in combination to recover both the contrasts and the total fields. Traditionally, (1) and (4) are used as they are to develop inverse scattering algorithms.

### 2.2. Integral Equations for Cavity *S*-Parameter Measurements

In a previous work [12], we showed that it is possible to transform (1) and (4) so that they are consistent with an *S*-parameter-based measurement system. We showed that the resulting equations were valid for both free-space and cavity-like geometries and went on to validate the free-space case with an inverse scattering experiment [13]. Here, we will summarize the results for a cavity geometry.

**Figure 1:**
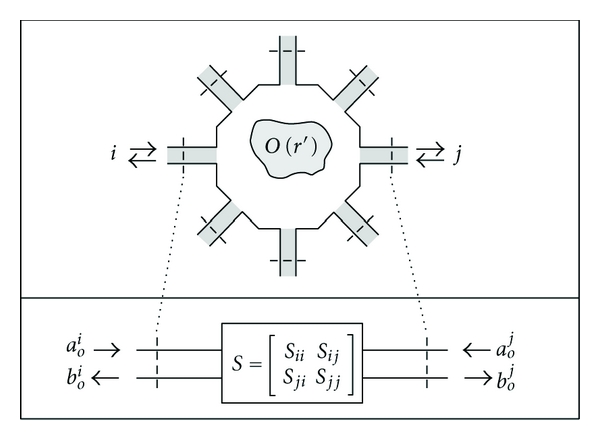
Microwave network model of cavity and scattering object. *S*-parameters are measured between the reference planes on the transmission lines.

Consider the cavity depicted in Figure 1. An object to be imaged is placed in the middle of the cavity. The cavity is filled with a background material having a permittivity and conductivity of *ϵ*_*b*_ and *σ*_*b*_, respectively. The cavity is lined with radiating apertures, which could be antennas. Each aperture has its own feeding transmission line and *S*-parameter reference plane.

We define the normalized incident and total fields throughout the cavity due to a transmitting aperture as

(6) $$\begin{array}{c} e_{\text{inc}}\left(r\right)=\frac{E_{\text{inc}}\left(r\right)}{a_{o}},\\ e\left(r\right)=\frac{E\left(r\right)}{a_{o}}, \end{array}$$

where *a*_*o*_ is the transmit voltage measured with respect to the *S*-parameter reference plane. The normalized incident field captures all background multiple scattering not present between the object and the cavity.

Let transmitting apertures be indexed with *i* and those receiving indexed with *j*. We can write (1) in terms of the normalized incident and total fields produced by a transmitter by dividing both sides by *a*_*o*,*i*_:

(7) $$e_{i}\left(r\right)=e_{\text{inc},i}\left(r\right)+\int \bar{G}\left(r,r^{\prime }\right)\cdot O\left(r^{\prime }\right)e_{i}\left(r^{\prime }\right)dV^{\prime }.$$

This is the integral equation we will use to represent the forward scattering solution. The normalized total field is the field solution in the object domain and, with the appropriate dyadic Green's function for the cavity, includes the scattering interactions between the object and the cavity.

In [12] we showed how to transform the scattered field volume integral equation given by (4) into one that predicts *S*-parameters. This new integral operator allows us to directly compare model predictions to measurements in the inversion algorithm. The two-port scattered field *S*-parameter, *S*_*ji*,sca_, measured between the transmission line reference planes of two apertures in the presence of an object is given by

(8) $$S_{ji,\text{sca}}=\int g_{j}\left(r\prime \right)\cdot O\left(r^{\prime }\right)e_{i}\left(r^{\prime }\right)dV^{\prime },$$

where **e**_*i*_(**r**) is the normalized total object field produced by the transmitter and **g**_*j*_(**r**) is the vector Green's function kernel for the receiver. It was also shown in [12] by reciprocity that **g**_*j*_(**r**) is related to the normalized incident field of the receiver as

(9) $$g_{j}\left(r\right)=-\frac{Z_{\text{o}}^{j}}{2i\omega \mu }e_{\text{inc},j}\left(r\right),$$

where *ω* is the operating frequency in radians, *μ* is the background permeability, and *Z*_*o*_^*j*^ is the characteristic impedance of the receiver transmission line.

Equations (7) and (8) are the integral equations we will use for the inverse scattering algorithm. They consistently link the electric field volume integral equations to an *S*-parameter measurement system. We need only to determine the normalized incident fields in the object domain and the background dyadic Green's function; no other step is required to characterize the system, except to calibrate the transmission line reference planes.

Lastly, in experiment, we never measure scattered field *S*-parameters directly but obtain them by subtracting the *S*-parameters for the total and incident fields:

(10) $$S_{ji,\text{sca}}=S_{ji,\text{tot}}-S_{ji,\text{inc}},$$

where *S*_*ji*,inc_ is measured in the absence of the object and *S*_*ji*,tot_ is measured in the presence of the object.

### 2.3. Determining **e**_inc_(**r**) and $\overset{̅}{G}(r,r^{\prime })$

The normalized incident field is required in both (7) and (9) and is required for every aperture. We can either measure it experimentally or estimate it with simulation. Experimentally mapping the fields requires proper probe calibration and has the added complication in a cavity that the probe-wall interactions cannot be neglected. An alternative approach, the one we adopt for this paper, is to estimate the normalized incident field with simulation. This can be done provided that we have a computer aided design (CAD) model that accurately represents the cavity. It is also possible in simulation to model the feeding transmission lines and line voltages in order to assign an *S*-parameter reference plane that is identical to the reference plane used by a vector network analyzer for the physical measurement. We will show how we use Ansoft HFSS to accomplish this.

As stated in the introduction, determining the background dyadic Green's function is nontrivial, especially for arbitrary cavity geometries. Despite this, for the immediate investigation, we use the free-space dyadic Green's function under the condition that the background medium is extremely lossy. Though not strictly correct, this approximation is convenient provided the multiple scattering throughout the cavity is limited by the background loss. It also allows us, for the time being, to retain use of an FFT-based volumetric forward solver. We give examples later evaluating this assertion. There are several approaches for determining or approximating the background dyadic Green's function for arbitrary geometries, which we discuss in The Appendix and leave for future work.

## 3. Born Iterative Method

The imaging algorithm we use is the Born iterative method (BIM) [16–19]. The BIM successively linearizes the nonlinear problem by alternating estimates of the contrasts and the object fields according to the following algorithm.

1. Assume the object fields are the incident fields (Born approximation).
2. Given the measured scattered field data, estimate the contrasts with the current object fields by minimizing a suitable cost function.
3. Run the forward solver with current contrasts. Store the updated object field.
4. Repeat step 2 until convergence.

This algorithm and its implementation are described in detail in our previous work [13], where we successfully formed images of dielectric constant for plastic objects in a free-space experiment. This was done using the same BIM and the integral equations for *S*-parameters given above using antennas characterized with HFSS.

We use the multivariate covariance-based cost function of [15]. The Gaussian interpretation of this cost function allows us to experimentally justify the values we use to regularize it by our *a priori* knowledge of the experimental noise and range of contrast values. For the forward solver, because we use the lossy free-space dyadic Green's function to model the internal scattering, we use the BCGFFT [20–22], which we have validated with analytic solutions. In the examples that follow, we found that 4 BIM iterations were repeatably sufficient for the data residual and object to converge.

## 4. Breast Imaging System Prototype

**Figure 2:**
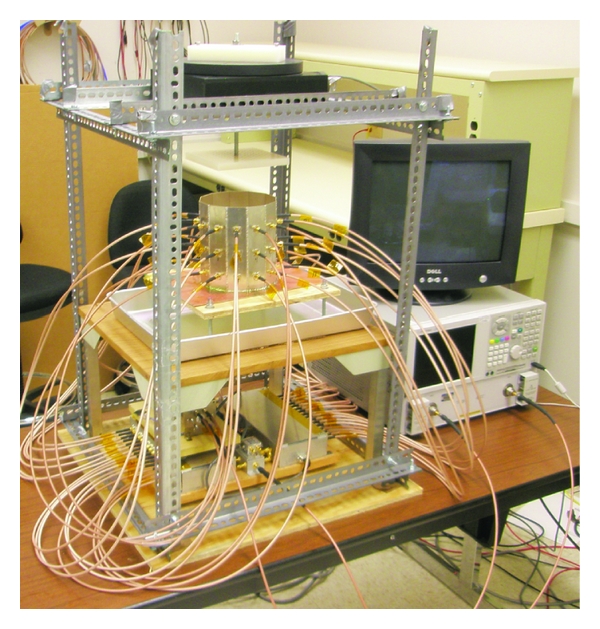
Breast imaging system prototype. The imaging cavity is connected to the VNA through a solid-state switching matrix. A rotator is mounted above and turns suspended objects for multiple transmitter views.

**Figure 3:**
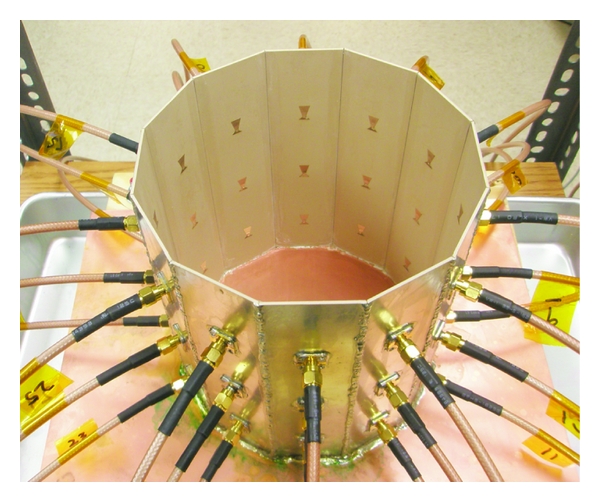
Imaging cavity. Twelve panels with three bow-tie antennas each are solder together and to a conducting plate.

The breast imaging system prototype we built is shown in Figure 2. The imaging structure is a cavity, shown in Figure 3, that was created by soldering twelve vertical panels of microwave substrate together and soldering the collection to a conducting base. Opposite panels are separated by 15 cm, and the cavity is 17 cm long. Three antennas are printed on each panel for a total of 36 antennas. In the prototype, the three antennas of one panel are used as transmitters, while all other antennas are receivers. The transmit antennas are switched with a Dowkey SP6T electromechanical switch. The receivers are connected through an SP33T solid-state switching matrix that was designed and assembled in-house. 2-port *S*-parameter measurements were taken with an Agilent PNA-5230A vector network analyzer (VNA) at 2.75 GHz between each transmitter and any one receiver. This frequency was chosen as a compromise between resolution and switch performance, which rolls off above 3 GHz. A rotator was mounted above the cavity and aligned in the center of the cavity. Test objects are suspended with fishline and rotated to provide multiple transmitter views.

### 4.1. Liquid Coupling Medium

We expect breast tissue to have a relative permittivity between 10 and 60 [23]. Without a matching medium, much of the incident power would be reflected at the breast/air interface reducing the sensitivity of the system [24]. Also, the contrast ratio between the object and the background would be too high for the BIM inverse scattering algorithm to converge.

The matching medium we use is an oil/water emulsion developed in a previous work [25]. This fluid is designed to balance the high permittivity and high conductivity of water with the low permittivity and low conductivity of oil, in order to achieve a fluid with moderate permittivity while limiting loss as much as possible. We are also able to tune the microwave properties of this emulsion by adjusting the oil/water ratio. We aimed for a relative permittivity value around 20, which brings the maximum permittivity contrast to about 3 : 1. The fluid mixture we used was 65%/35% oil/water.

The electrical properties of the fluid were measured using the Agilent 85070E slim form dielectric probe. The measured properties at 2.75 GHz were (*ϵ*_*r*_, *σ*) = (19,0.34). Relative permittivity is unitless; the units of conductivity used throughout the paper are Siemens/m. When using this value in the numerical model (presented below) the magnitude of cross-cavity *S*_21_ required some adjustment when compared to the measurements. We obtained the best model agreement for (*ϵ*_*r*_, *σ*) = (21,0.475), which are the values we use throughout the paper. We suspect that the probe area may be too small to accurately measure the bulk properties of the mixture, but the fluid otherwise appears homogeneous for propagation at 2.75 GHz. We are still investigating this effect.

When taking data, we fill the cavity with the coupling fluid to a height that is 0.5 cm below the top edge. This fluid height is accounted for in the numerical model. Any fluid displacement from adding or removing test objects is compensated in order to keep the height constant. We have also found the emulsion to be stable over the course of measurements, which we confirmed by comparing transmission measurements before and after we take data for imaging.

### 4.2. Antenna Design

The antennas are bow-tie patch antennas, similar to the antennas in [6, 26]. They are of single frequency and vertical polarization. The bow-tie antenna was chosen to give more degrees of freedom to help impedance match the antenna to the coupling fluid. The vertical polarization was chosen for best illumination of the object and other antennas in the cylindrical geometry. The substrate material is Rogers RO3210, with 50 mil thickness and reported dielectric constant of 10.2. The antennas were originally designed to operate at 2.8 GHz in the cavity filled with a fluid with (*ϵ*_*r*_, *σ*) = (24,0.34); however, after iterating, we found best performance at 2.75 GHz in a fluid of (*ϵ*_*r*_, *σ*) = (21,0.475).

### 4.3. System Parameters

In determining the system noise and isolation requirements, the minimum expected signal determines the required noise level, and the maximum relative magnitude between signals on adjacent channels determines the required switch path isolation. From previous numerical studies of cavity-like breast imaging with similar emulsion properties [27], we expect the scattered field *S*_21_ magnitude of small inclusions to be in the range from −100 to −50 dB, and so the relative signal strength between adjacent antennas could differ by as much as −50 dB. This means that the noise of our system must be less than −100 dB, which is achievable by our VNA with averaging and an IF bandwidth of 100 kHz or less. Also, the switching matrix paths must be isolated by at least −50 dB.

### 4.4. Switching Matrix

The receivers were connected through a SP33T solid-state switching matrix that was designed and assembled in-house. The matrix consists of two custom SP16T solid-state switching matrices and a cascaded pair of Miniciruits SPDT switches. Each SP16T switch is composed of two layers of SP4T Hittite HMC241QS16 nonreflective switches, which are buffered at the output by a third layer consisting of a single SPDT Hittite HMC284MS8GE on each path. The buffer layer was added to increase interpath isolation. The switch is controlled with an embedded digital board and computer parallel port. The operating band of the switching matrix is between 0.1–3 GHz. The overall loss of a path through the SP33T matrix is no worse than 8 dB across the band. We measured the switch path isolation to be better than −55 dB between 1–3 GHz, which meets the criteria above.

By separating the transmitter and receiver switching, the isolation between these two operation modes is dictated by the network analyzer and the cables. In more realistic systems, where the antennas are dual mode and so object rotation is not necessary, the isolation requirements are more stringent, because the transmit amplitude will be orders of magnitude larger than the scattered field.

### 4.5. VNA Calibration

Two-port VNA calibrations were accomplished between each transmitter and each receiver. The *S*-parameter reference planes were calibrated to the points where the cables connect to the antenna. These reference planes are identical to those in the HFSS CAD model (presented below). While calibrating, we left the unused ports open with the rationale that the one-way switch isolation of −55 dB provided sufficient matching to the open ports. Short-open-load measurements for a 1-port calibration were taken for each antenna. Next, we measured the through path between the transmitter and each receiver using a connector. In software, we combined the 1-port and through measurements to accomplish a 2-port short-open-load-through (SOLT) calibration with arbitrary through between each transmitter and receiver for a total of 99 separate 2-port calibrations. The calibration for a particular transmitter/receiver pair is recalled in the VNA before taking data.

## 5. Numerical Model

We use Ansoft HFSS to numerically model the cavity, similar to [27]. We use it several ways. First, we model the feeding transmission lines in order to assign *S*-parameter reference planes that are identical in both simulation and experiment. Second, we estimate the normalized incident fields due to the transmitters throughout the cavity for use in (7) and (9), where the normalized incident fields now include all background multiple scattering not present between the object and the cavity. Also, we use the model to generate synthetic scattered field *S*-parameters of numerical targets in order to study the performance of the inverse scattering algorithm given the source geometry and system parameters.

**Figure 4:**
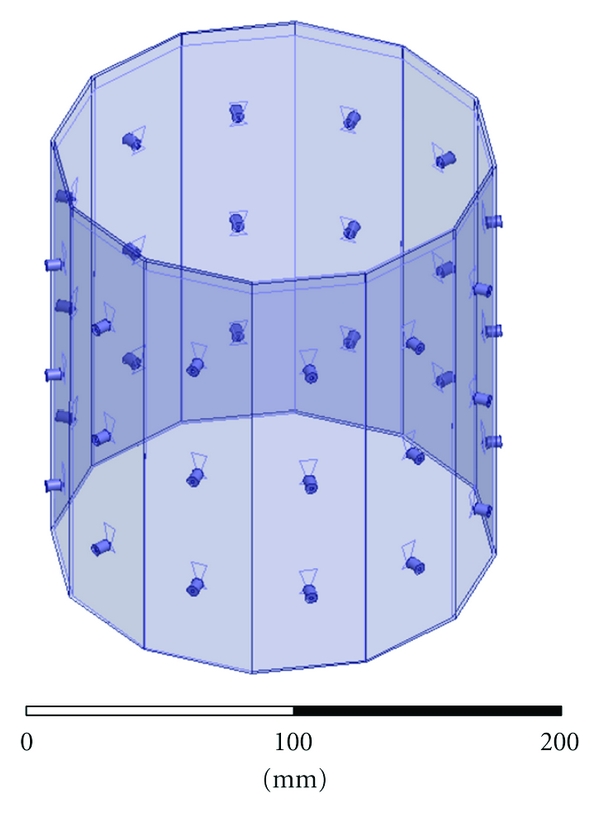
HFSS CAD model of the imaging cavity. Twelve panels contain three bow-tie antennas each. The bottom of the cavity is PEC, it is filled with the coupling fluid up to the visible line, and the top surface radiates to air.

Figure 4 shows the HFSS CAD model of the 12-sided cavity. The model includes the panel thickness and dielectric constant, bottom conductor, probe feed, coupling fluid properties, and height of the fluid. Same as in the experiment, the cavity is filled to a height that is 0.5 cm below the top (seen as the line below the top edge of the cavity). The remaining 0.5 cm is air with a radiating boundary condition. The outer boundary of the cavity is PEC.

**Figure 5:**
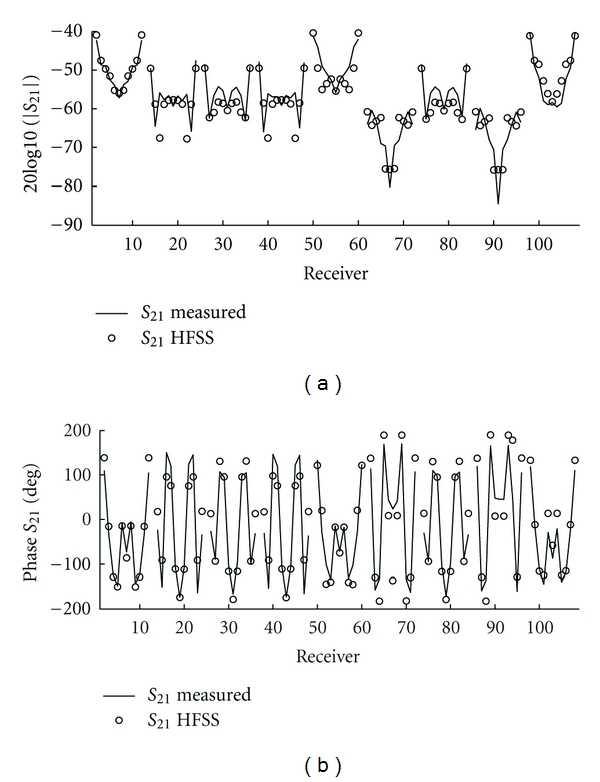
Measured and simulated magnitude and phase of incident *S*_21_ between each of the three transmitting antennas and all receivers. Solid: measured. Dots: HFSS. The groupings from left to right are the eleven receivers of each level (middle, top, and bottom), repeated for the three transmitters (middle, top, and bottom), plotted counterclockwise when viewed from above for a given receiver level. For example, data 38 : 48 are middle receivers and top transmitter. The magnitude and phase agree best for transmitters and receivers on the same level (i.e., data 1 : 11, 50 : 60, and 98 : 108).

Next we compare measured and simulated incident *S*-parameters in order to access the accuracy of the model.  Figure 5 shows the magnitude and phase, respectively, of the measured and simulated incident *S*-parameters between each transmitter and all receivers. The magnitude and phase agree best when the receivers are on the same level as the transmitter. In this case, the magnitude agrees generally to within 3 dB, for all three levels, and the phase agrees to within 30 degrees, which is approximately *λ*/10, a common metric for many microwave systems. For measurements between antenna levels in Figure 5, the agreement is not as good in magnitude, but the phase error remains similar to the previous cases. This also shows that the one-way path loss across the cavity is approximately −50 dB, so we expect any multiple scattering to be localized. This partially justifies our approximation of the cavity dyadic Green's function with the lossy free-space dyadic Green's function.

**Figure 6:**
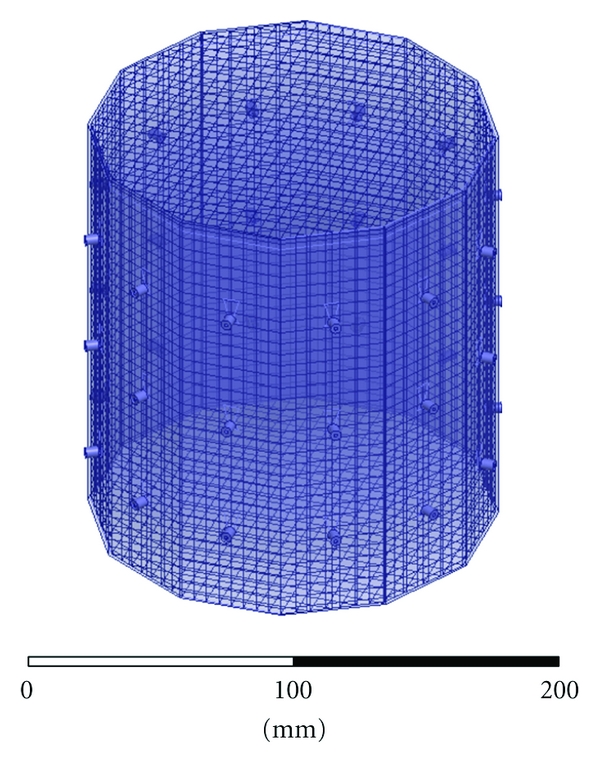
HFSS CAD model of the imaging cavity with mesh of unassigned sheets to constrain the adapting meshing of HFSS for field interpolation. Sheets are spaced every 5 mm in each direction.

When computing the incident fields, the center of the cavity was meshed with a coarse Cartesian grid of sparse unassigned sheets, shown in Figure 6. Sheets are spaced every 5 mm in the *x*, *y*, and *z* directions. The spacing is approximately *λ*/5 at 2.75 GHz in the fluid with relative permittivity of 21. We have found that this helps constrain the adaptive meshing of HFSS when we obtain the incident fields by interpolating the FEM mesh onto a fine Cartesian grid, [12].

**Figure 7:**
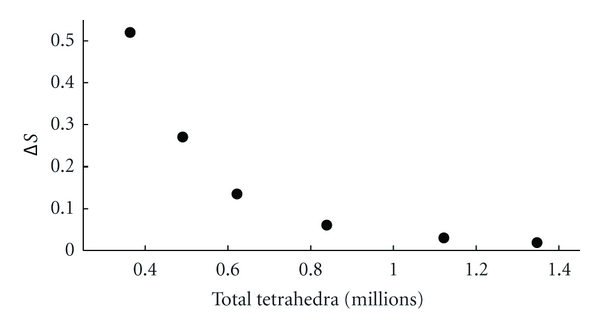
HFSS convergence with number of tetrahedra for each adaptive meshing step.

When simulating the structure, with or without scattering targets, we use a convergence criterion of Δ*S* = 0.02 which is reached in 7 adaptive meshing iterations. A typical simulation completed with approximately 1.4 million tetrahedra using 23.5 GBytes of RAM and swap space to obtain a full 36 × 36 *S*-matrix. Simulations took approximately 25 hours on a dual E5504 Intel Xeon (2x Quad Core) desktop with 24 GBytes of RAM.  Figure 7 shows a typical convergence rate as a function of tetrahedra.

We obtained the incident fields for only the three transmitters. The incident fields for the receivers were obtained through rotation, where we assume the 12 panels of the experimental cavity are identical. The incident fields were sampled on a 17 cm × 17 cm × 18 cm grid with 1 mm spacing, which is *λ*/24 at 2.75 GHz in a fluid with relative permittivity 21. In simulation, the average transmit power was 1 Watt, so, from transmission line analysis, the line voltage is given by

(11) $$a_{o}=\sqrt{2P_{\text{ave}}Z_{o}}=\sqrt{2Z_{o}},$$

which is used in (6). The phase of *a*_*o*_ is zero because the *S*-parameter reference planes of the HFSS model and the experimental cavity were identical.

**Figure 8:**
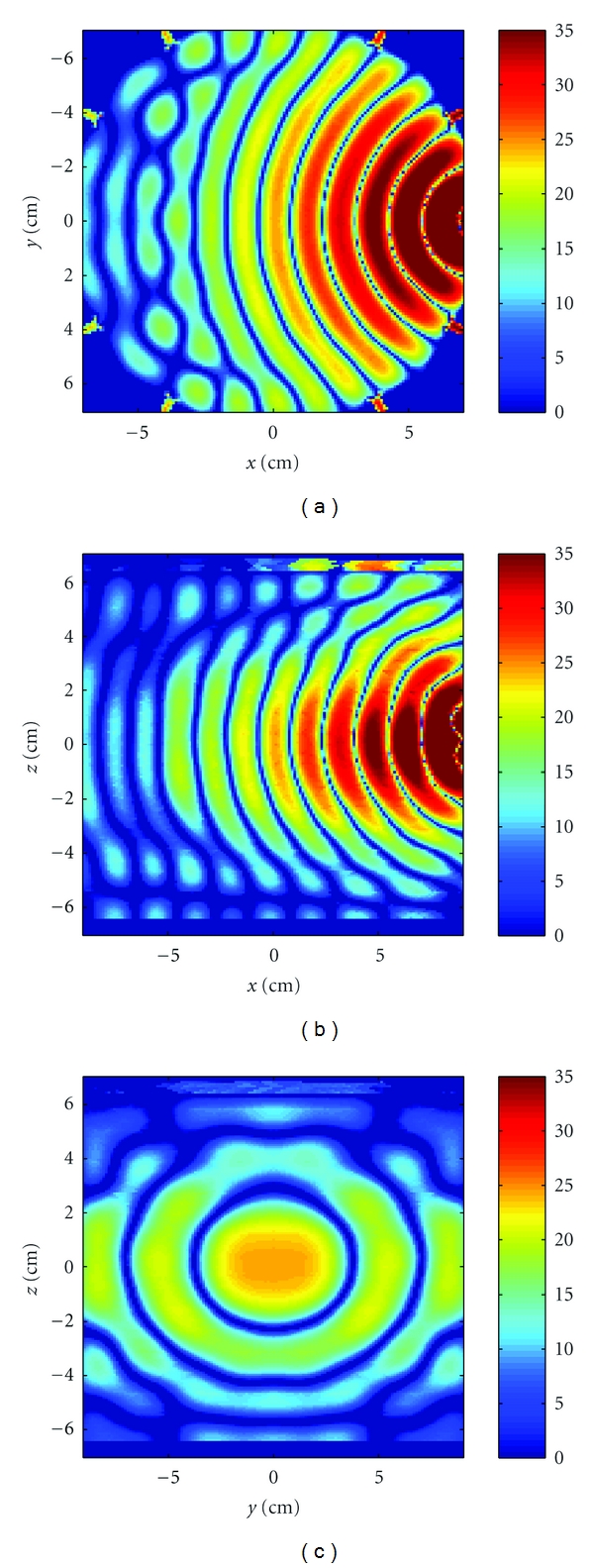
Crosscuts through the center of the cavity of the *z*-component of the incident electric field due to the middle transmitter. The scale is 20log10(|*Re*{*E*_*z*,inc_}|) of the unnormalized field. (a) Horizontal *x*-*y*, and (b) vertical *x*-*z*, and (c) vertical *y*-*z* planes.

Figure 8 shows three crosscuts of the *z*-component of the incident electric field through the center of the cavity for the center transmitter in a fluid of relative permittivity of 21 and conductivity 0.475 at 2.75 GHz. The coordinate origin is at the center of the cavity, and the transmitter is located on the positive *x* axis. The effects of the cavity on the incident field are seen in Figure 7, where the fields are guided by the walls of the cavity; the coaxial feeds are also visible; the fluid-air interface is visible in Figures 8(b) and 8(c).

## 6. Image Reconstructions

### 6.1. Synthetic Data

We first test the BIM and numerical characterization using synthetic data from HFSS. This is to assess the performance of the algorithm and source geometry under near ideal circumstances. We simulated the scattered field *S*-parameters of simple numerical objects and use these data as measurements in the inversion algorithm. HFSS scattered field data includes any multiple scattering between the object and the cavity. The background medium had a relative permittivity of 21 and a conductivity of 0.475 Siemens/m. The incident fields were computed with these background parameters and used in volume integral equations.

**Example 1**

**Figure 9:**
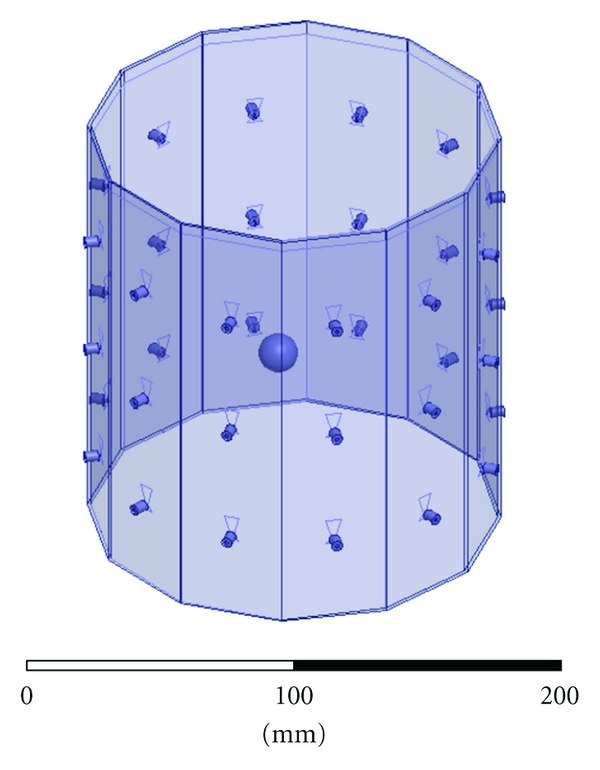
HFSS model of a simple sphere used to generate synthetic scattered field *S*-parameters.

**Figure 10:**
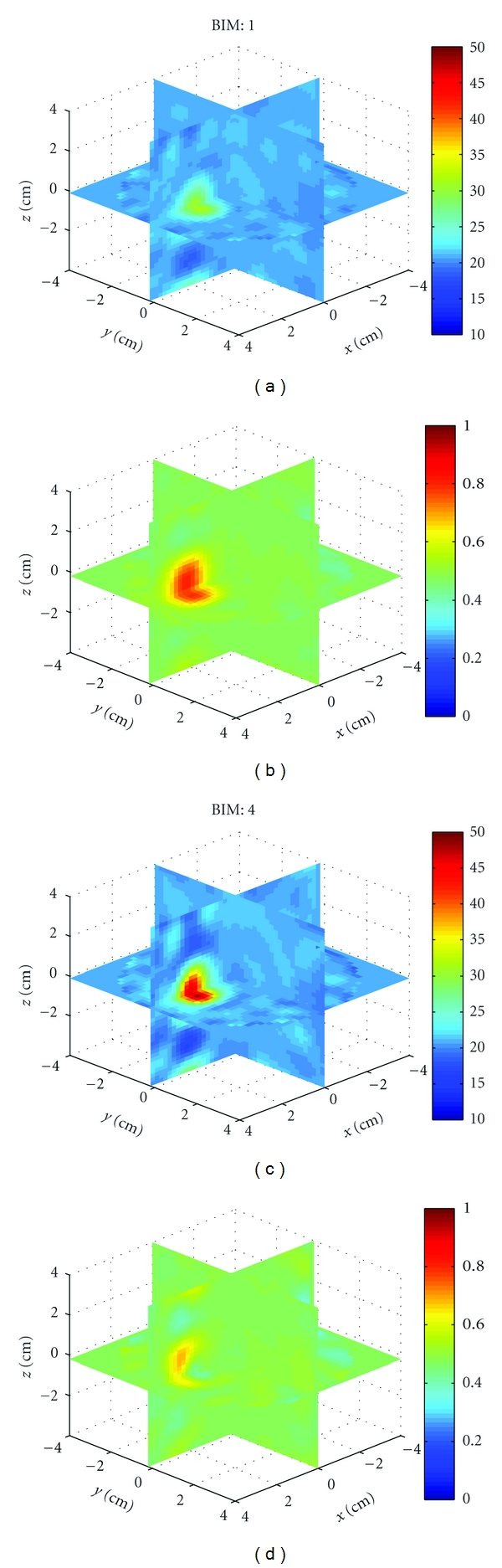
Reconstructions of a single sphere (*ϵ*_*r*_, *σ*) = (40, 1) located at (*x*, *y*, *z*) = (0,0, 2 cm) of Example 1. (a) and (c) and (b) and (d) Relative permittivity and conductivity, respectively. (a) and (b) is the Born approximation. (c) and (d) is BIM iteration 4. Here, iterations help retrieve the relative permittivity in (c), but the Born approximation yielded better conductivity in (b).

**Figure 11:**
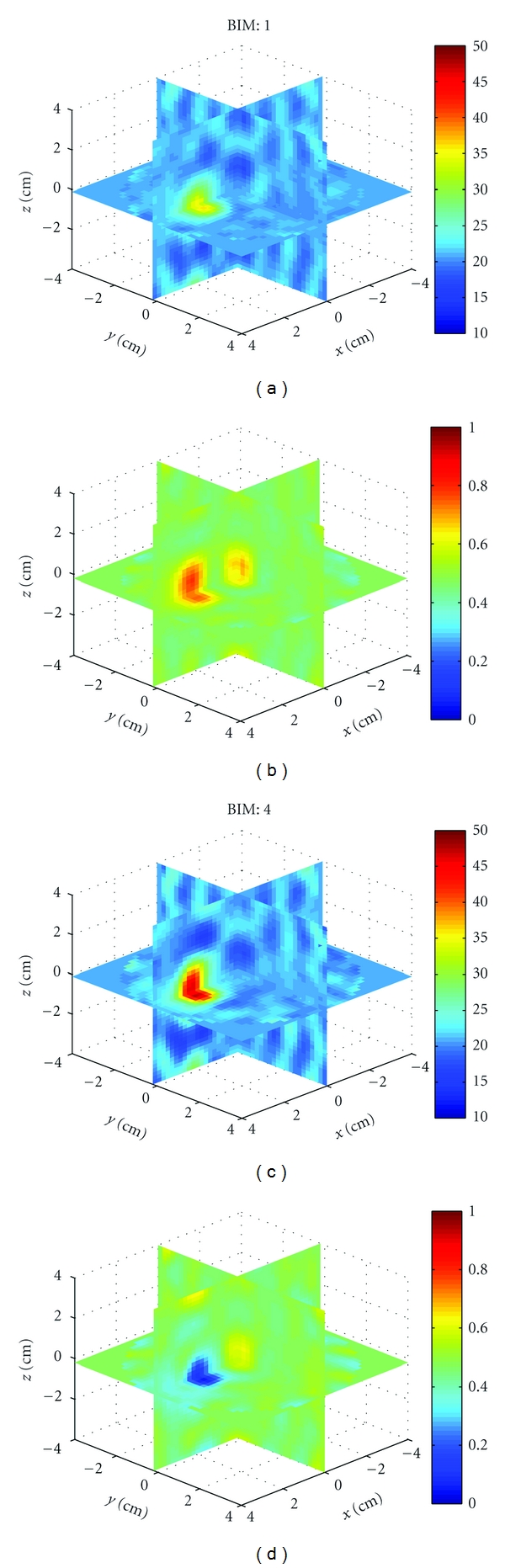
Reconstructions of a single sphere (*ϵ*_*r*_, *σ*) = (40, 0) located at (*x*, *y*, *z*) = (0,0, 2 cm) of Example 1. Born iterations helped retrieve the relative permittivity in (c) and are essential in recovering the conductivity in (d).

**Figure 12:**
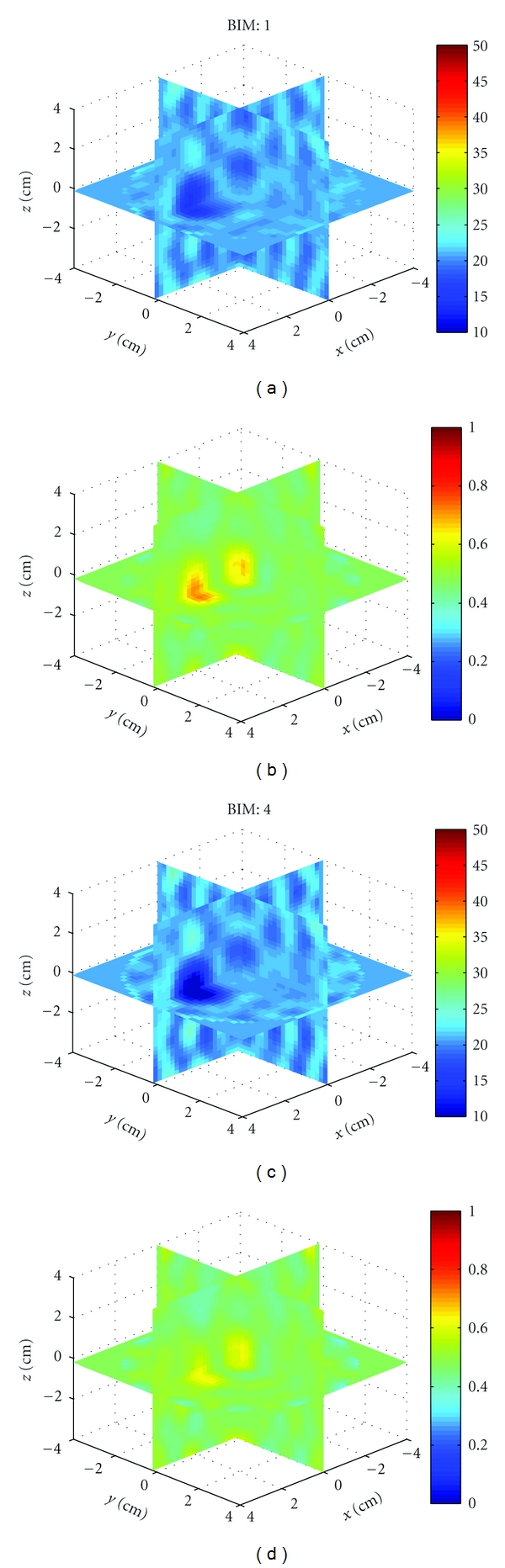
Reconstructions of a single sphere (*ϵ*_*r*_, *σ*) = (10, 1) located at (*x*, *y*, *z*) = (0,0, 2 cm) of Example 1. Born iterations helped the recovery of the low permittivity in (c), but at the expense of the correct conductivity value which was better with the Born approximation in (b).

**Figure 13:**
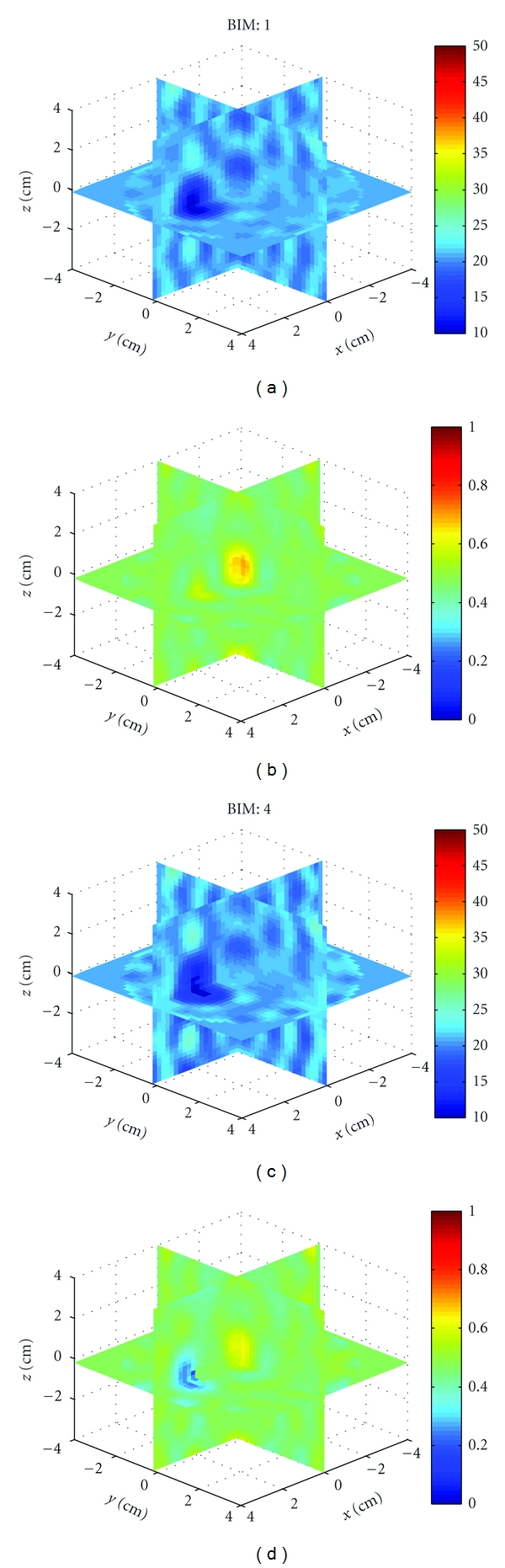
Reconstructions of a single sphere (*ϵ*_*r*_, *σ*) = (40, 0) located at (*x*, *y*, *z*) = (0,0, 2 cm) of Example 1. Born iterations helped bring out the proper conductivity value in (d).

We first used HFSS to simulate the scattered field *S*-parameters for a single 1.5 cm diameter sphere located at (*x*, *y*, *z*) = (0,0,2 cm) with four combinations of relative permittivity and conductivity: (40, 1), (40, 0), (10, 1), and (10, 0). The HFSS model is shown in Figure 9. Figures 10, 11, 12, and 13 show images of the first and fourth BIM iterations for each object. As shown, in some cases, the BIM steps were essential in recovering the correct property values of the sphere; in other cases, the relative permittivity was improved at the expense of the conductivity value. These images show that the source geometry and numerical characterization are adequate for the retrieval of some object property combinations, but not others. This fact, together with the visible artifacts, suggests that the images could be improved with a denser source geometry.

**Example 2**

**Figure 14:**
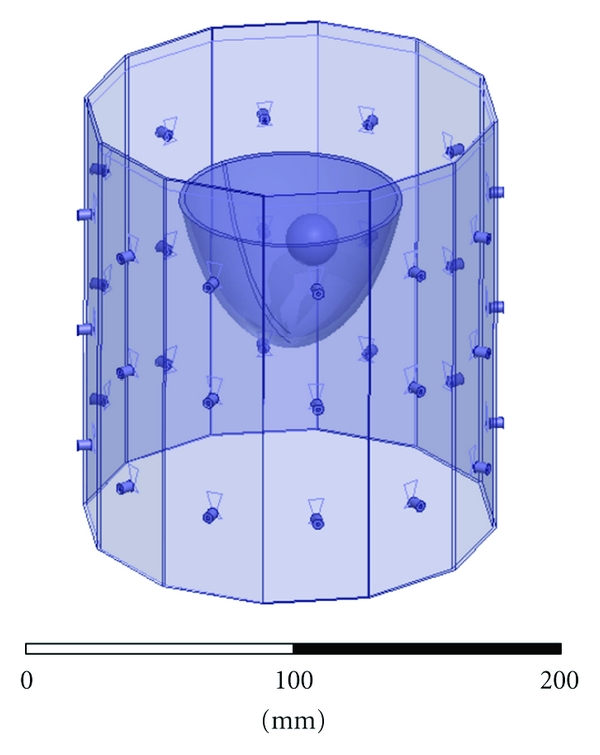
HFSS CAD numerical breast phantom of Example 2. The inclusion is 2 cm in diameter with relative permittivity and conductivity contrasts of 2 : 1. The skin layer is 2 mm thick.

**Figure 15:**
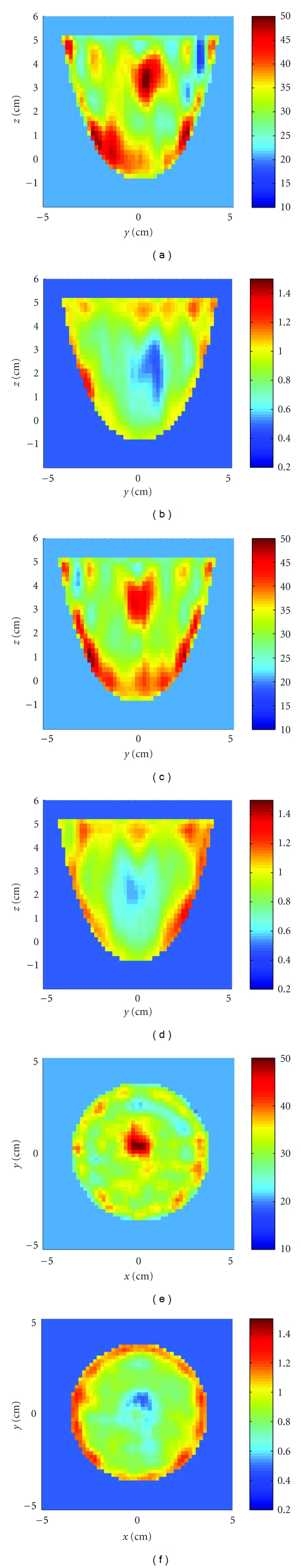
Reconstructions of the HFSS numerical breast phantom in Example 2 after four iterations. (a), (c), and (e) and (b), (d), and (f) Relative permittivity and conductivity, respectively. (a) and (b), (c) and (d), and (e) and (f) Cuts at *x* = 0 cm, *y* = 0 cm, and *z* = 3 cm. The relative permittivity of the inclusion is recovered, but both images contain many artifacts.

Next we imaged a more anatomical numerical breast phantom. The numerical phantom is shown in Figure 14. The breast is 9 cm at the widest point and 6 cm deep. The outer layer is a 2 mm thick skin layer, and the inclusion is 2 cm in diameter. The dielectric properties of the skin layer, glandular tissue, and inclusion, respectively, are (*ϵ*_*r*_, *σ*) = {(45,1.59), (21, 0.475), and (42, 0.8)}, which were obtained from [28]. We assume we know the volume region of the breast, so we mask that volume excluding all other points during inversion.  Figure 15 shows the reconstructed relative permittivity and conductivity after 4 iterations for three cuts. The relative permittivity of the inclusion is recovered, but the conductivity of the inclusion is not recovered. The skin layer is also visible in the conductivity images. Both sets of images suffer from artifacts, which is due to the sparse spatial sampling of the antennas and indicates that the images can be improved with more angular views.

**Example 3**

**Figure 16:**
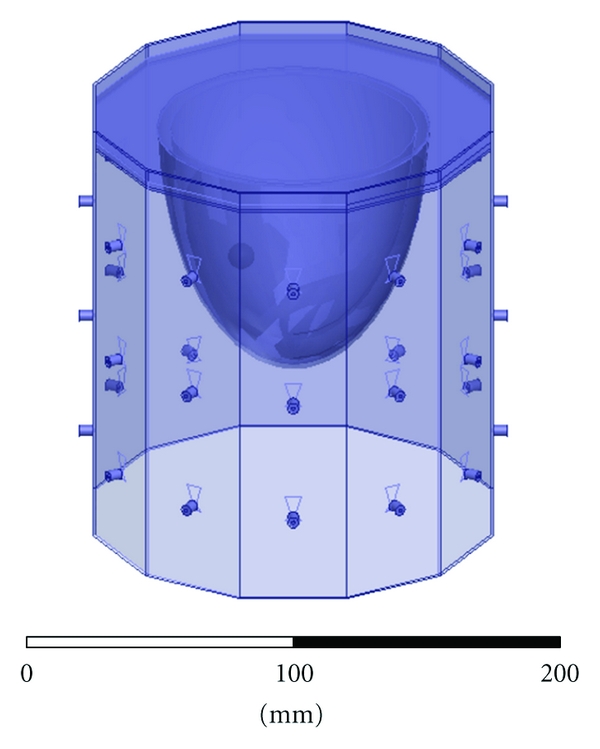
HFSS CAD numerical breast phantom with skin layer, fat layer, glandular tissue, and chest wall of Example 3. The inclusion is 1 cm in diameter with relative permittivity and conductivity contrasts of 2 : 1.

**Figure 17:**
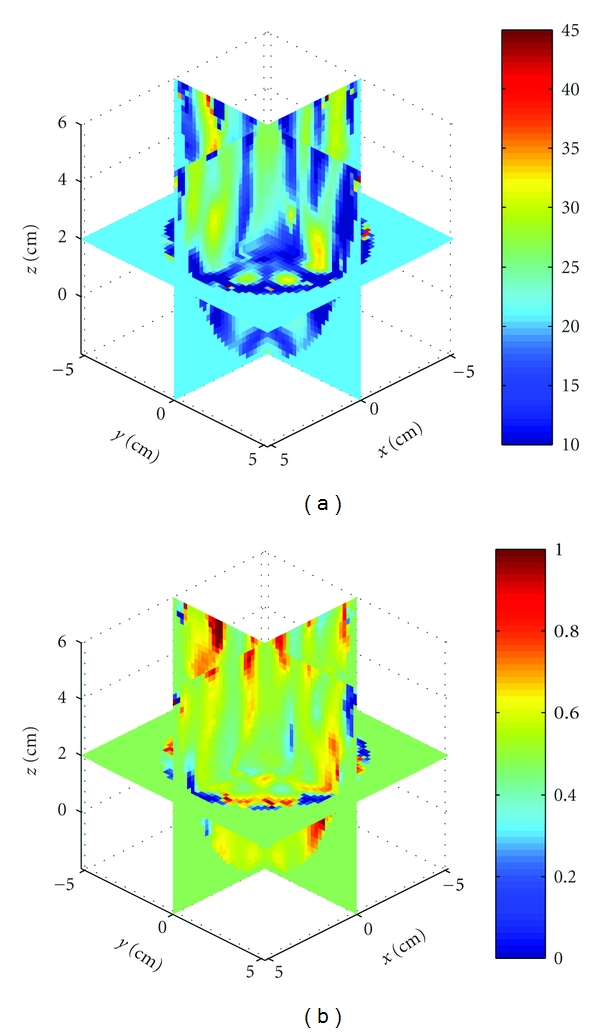
Reconstructions of the HFSS numerical breast phantom, which includes the chest wall of Example 3. (a) and (b) Relative permittivity and conductivity, respectively. The object could not be reconstructed.

To push the algorithm, we imaged a phantom that included a skin layer, fat layer, glandular tissue, chest wall, and inclusion, with relative permittivity and conductivity, respectively, of (45,1.6), (5.1,0.16), (21,0.475), (52, 2.0), and (40, 1.0). The HFSS model is shown in Figure 16. The reconstructions are shown in Figure 17. In this case the algorithm failed to recover the contrasts. This suggests that (1) the object is too different from the background for the BIM to converge, (2) object-cavity interactions are too strong to use the free-space dyadic Green's function, or (3) images cannot be constructed if the chest wall is not modeled, meaning that it is necessary to model the chest wall for the incident fields and the dyadic Green's function.

**Example 4**

Finally, we studied the effects of different background permittivities when forming images. This represents a case in experiment where the measurements are taken in a fluid with some set of properties, but the fluid properties we use in the model are slightly off. We formed images using HFSS scattered field data of the sphere with (*ϵ*_*r*_, *σ*) = (40, 0) in a background of (*ϵ*_*b*_, *σ*) = (21, 0.475), but where we use incident fields from five different background permittivities: {20, 20.5, 21 (again), 21.5, 22} and the same conductivity.

**Figure 18:**
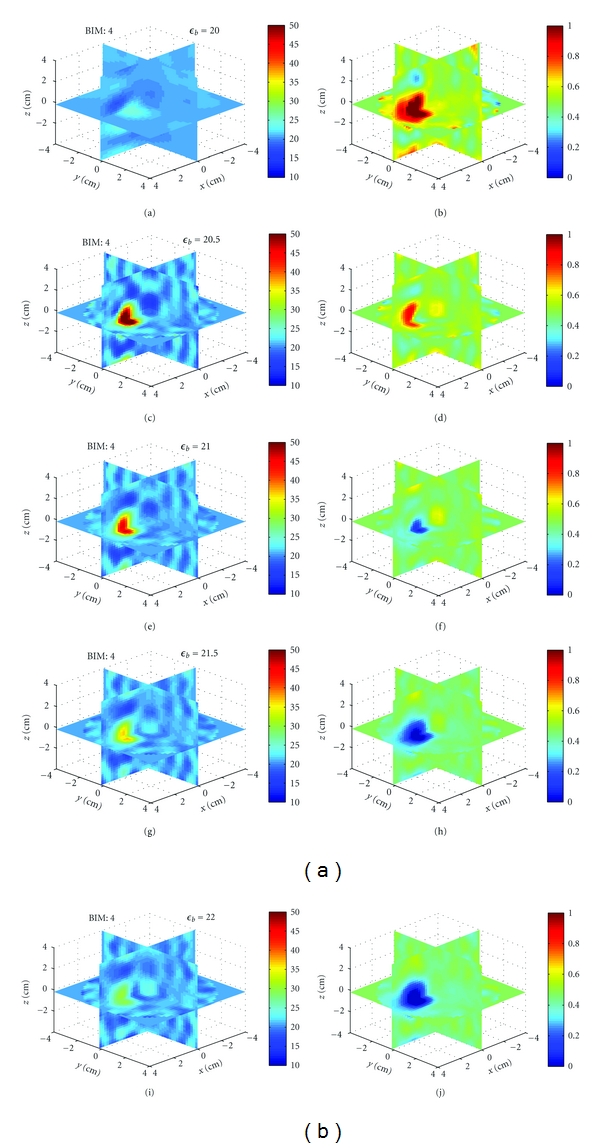
Sensitivity of image reconstructions to background permittivity for Example 4. (a), (c), (e), (g), and (i) and (b), (d), (f), (h), and (j) Relative permittivity and conductivity, respectively. The scattered field data was generated in HFSS in a background of (21, 0.475). The reconstructions are done with assumed background relative permittivities of {20, 20.5, 21, 21.5, 22} for (a) and (b), (c) and (d), (e) and (f), (g) and (h) and (i) and (j), respectively. The recovered contrasts of the sphere oscillate about the background.

Figure 18 shows 3D crosscuts at the fourth BIM iteration for all five backgrounds. Figures  18(e) and  18(f) are the correct images. Notice that an error in the background permittivity of 1, or 5%, is enough for the reconstructed object contrast to oscillate, demonstrating that reconstructions are very sensitive to our knowledge of the background properties.

### 6.2. Experimental Data

At this time, only simple plastic objects have been imaged with the experimental system; however, future work includes imaging more realistic breast phantoms. Among the test objects, we show the results here for several acrylic spheres. The objects were suspended from a platform and rotated to 12 positions in 30 degree increments. Scattered field *S*-parameter measurements from each position were combined to yield a full 36 × 36 *S*-parameter matrix, which was used in the inverse scattering algorithm.

**Experiment 1**

**Figure 19:**
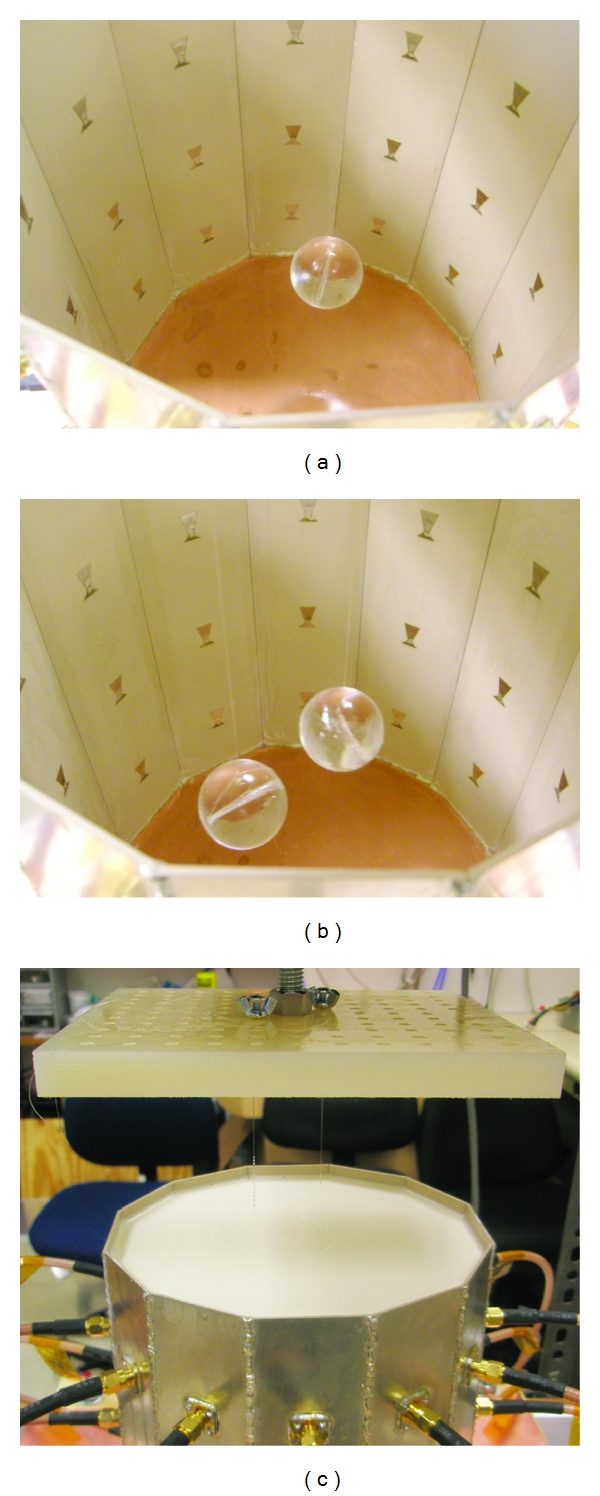
Test objects and coupling fluid for Experiment 1. (a) Single suspended acrylic sphere. (b) Two acrylic spheres. (c) Cavity filled with the coupling medium. Objects are suspended and rotated from the nylon platform.

**Figure 20:**
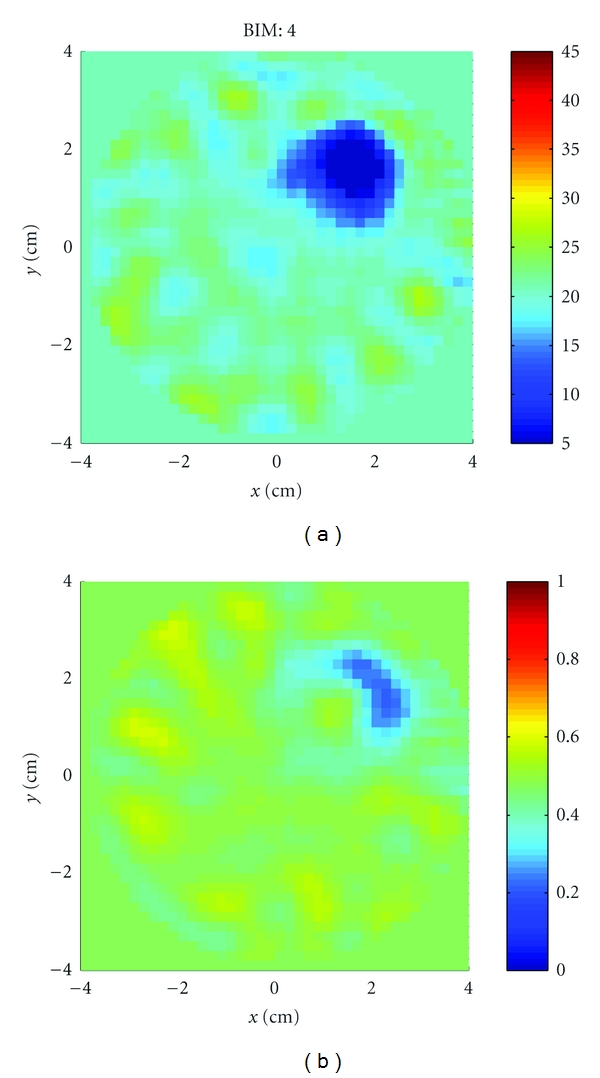
Reconstructions of the single acrylic sphere of Experiment 1 shown in Figure 19(a). (a): Relative permittivity. (b): Conductivity. The permittivity is recovered well but the shape in the conductivity is not.

**Figure 21:**
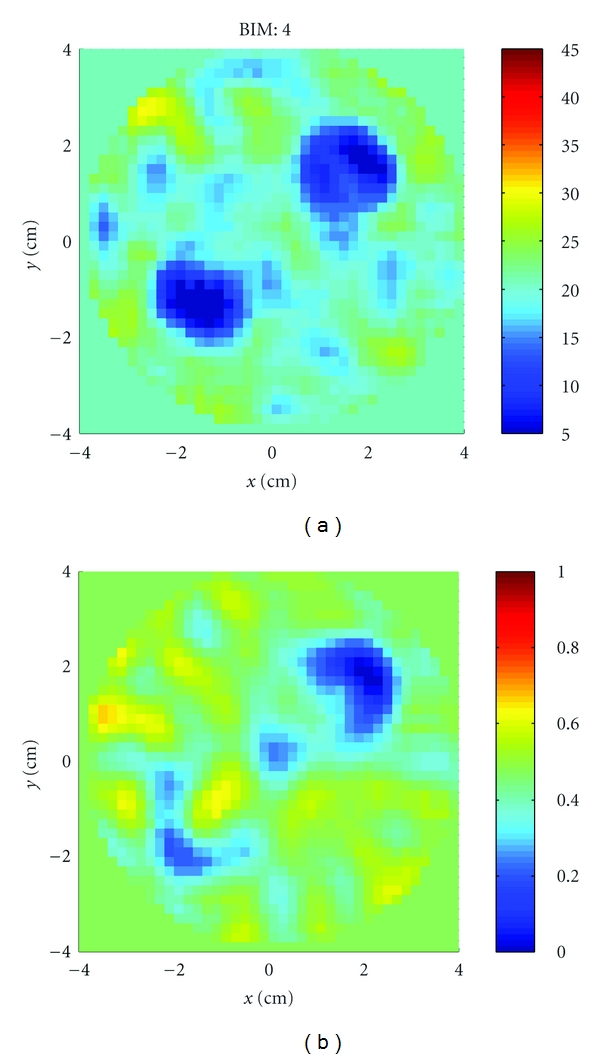
Reconstructions of the two acrylic spheres of Experiment 1 shown in Figure 19(b). (a) Relative permittivity. (b) Conductivity. The permittivity is again recovered well but the shape in the conductivity is not.

We imaged a single acrylic sphere, shown in Figure 19. The diameter of the sphere was 2.54 cm, with properties (*ϵ*_*r*_, *σ*) = (2.7, 0). The sphere was located at approximately (*x*, *y*, *z*) = (1.5 cm, 1.5 cm, 0).  Figure 20 shows the reconstructions after 4 iterations of the *x*-*y* plane. The inversion domain is masked so that only a cylindrical region containing the rotated object is imaged. We also imaged two acrylic spheres, shown in Figure 19.  Figure 21 shows the reconstructions after 4 iterations. In both cases, the relative permittivity is recovered quite well, and the conductivity contrast is correctly valued but the shape is incorrect. There are also many artifacts present. Given that the imaging algorithm could recover the single sphere using HFSS data, we can attribute these discrepancies to differences between the experiment and the model, such as knowledge in the coupling medium properties, substrate properties, VNA calibration, cavity size measurements, or object motion.

**Experiment 2**

**Figure 22:**
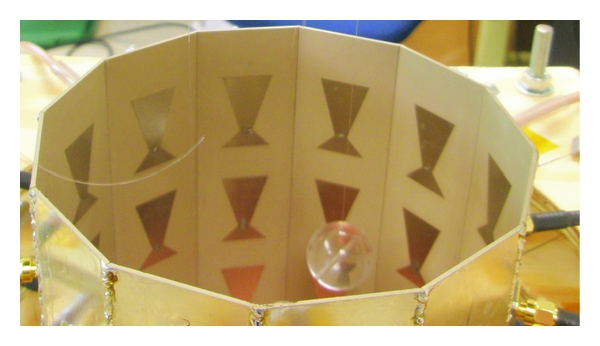
Second cavity with antennas designed to operate at 915 MHz of Experiment 2. Three acrylic sphere are suspended (one visible). Cavity is filled with fluid for imaging.

**Figure 23:**
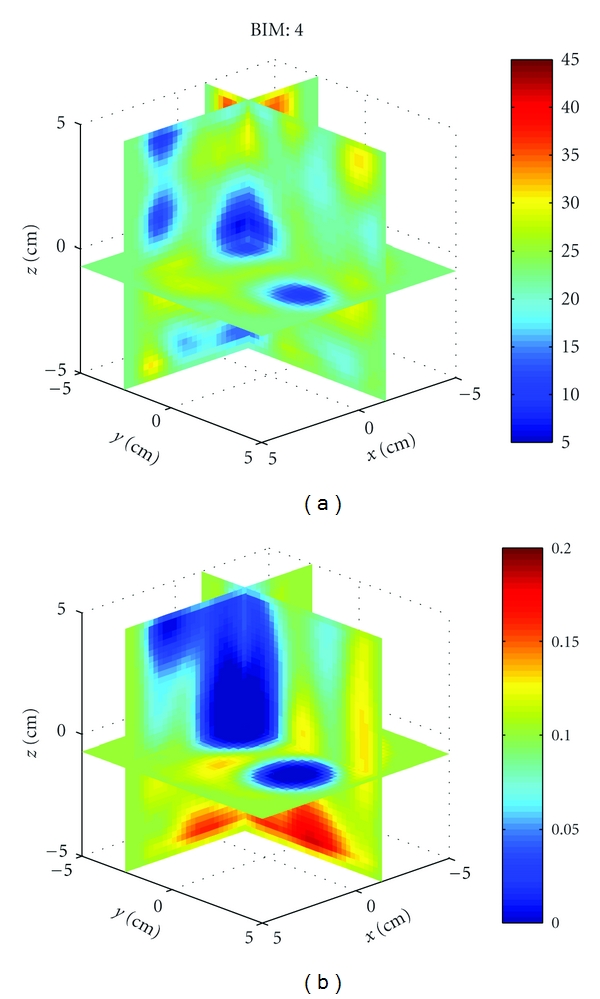
Reconstruction of the relative permittivity and conductivity of three acrylic spheres using a cavity operating at 915 MHz from Experiment 2. The two spheres in plane are well recovered and the third detected at the upper left of the image.

Finally, while the primary discussions in this paper concern a cavity having antennas that operate at 2.75 GHz, we also built a lower frequency cavity where the antennas operate at 915 MHz. This cavity was numerically characterized using the same methods, but the background fluid properties were (*ϵ*_*r*_, *σ*) = (23, 0.1).  Figure 22 shows the cavity with three acrylic spheres. Two spheres are located in the *x*-*y* plane, while the third is positioned at approximately (*x*, *y*, *z*) = (4 cm, −3 cm, 5 cm). We imaged the relative permittivity and conductivity, and the results after 4 iterations are shown in Figure 23. The shape and properties of the two in-plane spheres are well recovered. The third sphere is also detected but cut off at the upper left of the imaging domain. Artifacts are also present, but this example better demonstrates that the numerical characterization, BIM, and free-space Green's function are capable of recovering objects in this cavity and source geometry. It should be noted that images formed with data at 915 MHz are less susceptible to modeling errors because the cavity and objects are electrically smaller, but the resolution is reduced.

### 6.3. Discussion

Overall, the imaging algorithm, numerical characterization, and experiment worked with some success, and there are several areas for continued investigation.

First, Examples 1 and 2, and also Experiments 1 and 2, validate the technique described in this paper showing that the numerical characterization of the cavity incident fields and the use of the vector Green's function formulation linking the incident fields to the inverse scattering algorithm can be used to successfully form images in a cavity geometry. Examples 1 and 2 demonstrate the consistency of the method using synthetic scattered field *S*-parameter data. Experiments 1 and 2 show that the characterization and experiment agreed enough for the BIM to recover the location and permittivity of the test objects. More realistic phantoms and lower contrast phantoms will help further confirm the methodology.

Second, in Example 1, although some permittivity and conductivity combinations of the sphere were recovered, others were not. Given that the data was synthetic, this points to inherent imaging ambiguities in the simultaneous retrieval of both permittivity and conductivity in the inverse scattering problem. Possible solutions are increasing the number of unique data, or including prior information about the relations between permittivity and conductivity in tissue.

Third, the success of the algorithm in Example 2 in recovering the partial breast phantom suggests that our use of the lossy free-space dyadic Green's function in the forward solver of the BIM did not grossly affect image reconstruction in this case. This is keeping in mind that the synthetic scattered field *S*-parameter data did include any multiple scattering between the phantom and the cavity.

Fourth, in light of the successful reconstruction of the simple phantom in Example 2, the failure of the algorithm to recover the more complete breast phantom in Example 3 points to the need to model the chest wall. This can be done by including it in the incident field computations but it may also be necessary in estimating the cavity dyadic Green's function. This is an area to be investigated.

Lastly, Example 4 shows that we must know the background relative permittivity to within 5% of the actual or else risk incorrectly estimating whether the contrasts are higher or lower than the background. An equivalent error can arise from a correct background permittivity but incorrectly measuring the dimensions of the cavity. We suspect that the very high recovered conductivity values in both Experiments 1 and 2 may be due in part to these types of systematic errors. This demonstrates the difficulty in achieving the necessary consistency between the model, experiment, characterization, and imaging algorithm to accurately form microwave breast images of diagnostic quality.

## 7. Conclusion

We demonstrated the use of a numerical characterization technique for a breast imaging system prototype. We used HFSS to numerically estimate the incident fields of the antennas in a cavity geometry and formally linked them to an *S*-parameter-based inverse scattering algorithm and experimental setup. The imaging algorithm was the Born Iterative Method and recovered both numerical and experimental test objects with some success. Future work includes further validation of our methodology, imaging realistic breast phantoms, investigating practical solutions to modeling breast-cavity scattering interactions, image quality assessments with and without numerical characterization, and developing a hemispherical cavity and clinical imaging system.

## Appendix

### Determining the Background Dyadic Green's Function

We list the following approaches for obtaining the background dyadic Green's function as work for future investigation.

*Analytical Dyadic Green's Function*. There exist analytical solutions of the dyadic Green's function for some simple cavity geometries, such as cubes or cylinders [29], which might approximately model certain cavity-based imaging setups. These solutions, however, will likely not include finer details such as antenna plating, connectors, substrate material, or open-ended cavities, such as those used for breast imaging. Analytic solutions though lend themselves to the possibility of retaining some convolution structure in the VIE so fast forward solvers can be used (e.g., fast half space solutions [30], applied to multisided cavities). Determining the dyadic Green's function analytically becomes a formidable task as the geometry complicates, where simulation may be better suited.

*Full Numeric Simulation*. The most complete solution is to fully simulate the object and cavity using a numeric simulator, which will capture all the multiple scattering between the object and the cavity. However, unlike a dyadic Green's function, which only needs to be found once for a particular geometry and the values of which are only needed on the interior of the object domain, this method must simulate the cavity structure outside the object domain in every instance of the simulation. When used in an inverse scattering algorithm, which might compute the domain VIE for each source, frequency, and iteration, then repeatedly simulating the cavity structure adds to the already high computational burden. In addition, one must choose a proper simulation technique to handle both antenna surfaces and inhomogenous media.

*Numerical Dyadic Green's Function*. If analytical solutions are not accurate enough, then one must determine the dyadic Green's function numerically. This requires simulating three orthogonal dipoles in turn at every point in the object domain and recording the response at every other point in the domain. The dyadic Green's function is symmetric, so half of the combinations are redundant, and while the convolution nature of the VIE is destroyed, some computational speed-up is possible for symmetric operators. This technique, however, requires accurate modeling around the dipole singularity, which can be difficult. In the case of PEC structures, the technique in [31] computes the dyadic Green's function by finding an array of image dipoles outside the cavity, which avoids the complications from the singularity. The main advantage of determining the dyadic Green's function numerically is that, once found, we no longer need to simulate the cavity structure and can turn our attention to optimizing the computation of the dyadic Green's function.

*Approximate Solutions*. If the background loss is sufficiently high, so that the resonances of the cavity are damped, then we can approximate the dyadic Green's function. This can be done by adopting an analytical solution (e.g., free-space or cavity) or by, for instance, developing a perturbation method. Adopting the free-space dyadic Green's function (or a perturbation on it) also allows us to retain the convolution structure of the VIE- and FFT-based forward solvers, which may be more beneficial to the inverse scattering algorithm than modeling higher-order multiple scattering.
